# Digital Health Equity and Tailored Health Care Service for People With Disability: User-Centered Design and Usability Study

**DOI:** 10.2196/50029

**Published:** 2023-11-28

**Authors:** Sandeul Ha, Seung Hee Ho, Young-Hyeon Bae, Minyoung Lee, Ju Hee Kim, Ju Han Kim, Jisan Lee

**Affiliations:** 1 Department of Nursing Kyung-Hee University Medical Center Seoul Republic of Korea; 2 Rehabilitation Research Institute Korea National Rehabilitation Center Seoul Republic of Korea; 3 Department of Holistic Integrative Healing Studies Seoul Cyber University Seoul Republic of Korea; 4 College of Medicine Seoul National University Seoul Republic of Korea; 5 Department of Nursing Gangneung-Wonju National University Wonju, Gangwon State Republic of Korea

**Keywords:** digital health equity, digital health care service, COVID-19, mobile health, mHealth, mobile apps, needs assessments, heuristic, people with disability, caregivers, health personnel, mobile phone

## Abstract

**Background:**

As digital health services advance, digital health equity has become a significant concern. However, people with disability and older adults still face health management limitations, particularly in the COVID-19 pandemic. An essential area of investigation is proposing a patient-centered design strategy that uses patient-generated health data (PGHD) to facilitate optimal communication with caregivers and health care service providers.

**Objective:**

This study aims to conceptualize, develop, and validate a digitally integrated health care service platform for people with disability, caregivers, and health care professionals, using Internet of Things devices and PGHD to contribute to improving digital health equity.

**Methods:**

The methodology consists of 5 stages. First, a collaborative review of the previous app, Daily Healthcare 1.0, was conducted with individuals with disabilities, caregivers, and health care professionals. Secondly, user needs were identified via personas, scenarios, and user interface sketches to shape a user-centered service design. The third stage created an enhanced app that integrated these specifications. In the fourth stage, heuristic evaluations by clinical and app experts paved the way for Daily Healthcare 2.0, now featuring Internet of Things device integration. Conclusively, in the fifth stage, an extensive 2-month usability evaluation was executed with user groups comprising individuals with disabilities using the app and their caregivers.

**Results:**

Among the participants, “disability welfare information and related institutional linkage” was the highest priority. Three of the 14 user interface sketches the participants created were related to “providing educational content.” The 11 heuristic evaluation experts identified “focusing on a single task” as a crucial issue and advocated redesigning the home menu to simplify it and integrate detailed menus. Subsequently, the app Daily Healthcare 2.0 was developed, incorporating wearable devices for collecting PGHD and connecting individuals with disabilities, caregivers, and health care professionals. After the 2-month usability evaluation with 27 participants, all participants showed an increase in eHealth literacy, particularly those who used the caregiver app. Relatively older users demonstrated improved scores in health IT usability and smartphone self-efficacy. All users’ satisfaction and willingness to recommend increased, although their willingness to pay decreased.

**Conclusions:**

In this study, we underscore the significance of incorporating the distinct needs of individuals with disabilities, caregivers, and health care professionals from the design phase of a digital health care service, highlighting its potential to advance digital health equity. Our findings also elucidate the potential benefits of fostering partnerships between health consumers and providers, thereby attenuating the vulnerability of marginalized groups, even amid crises such as the COVID-19 pandemic. Emphasizing this imperative, we advocate for sustained endeavors to bolster the digital literacy of individuals with disabilities and champion collaborative cocreation, aiming to uphold the collective ethos of health and digital health equity.

## Introduction

### Background

According to the definition provided by the World Health Organization, health equity is the absence of unfair, avoidable, and remediable differences in health status among people and is achieved when everyone can attain their full health potential [1]. A subset of this, digital health equity is the readiness of all individuals to access digital health, regardless of age, race, income, or technology access, ensuring that no one is left behind due to a lack of connectivity or literacy [2]. In this era of digital proliferation, discrepancies in the accessibility, capacity, and quality of health information technology pose significant challenges. Researchers, including UNICEF, have advocated literacy-related studies and education targeting vulnerable populations to overcome the digital divide [3,4]. However, the digital divide persists. The imperative to overcome this digital divide and enhance digital health equity arises from its contribution to health inequity, posing a threat to overall health equity. To promote an inclusive digital society, digital health interventions should prioritize health equity and accessibility for those with disabilities, ensuring that they do not exacerbate inequalities while leveraging technology’s potential to overcome physical barriers and enhance health equity [5].

People with disability require more focused and continuous symptom management. According to the American Association on Health and Disability’s COVID-19 and Disability Survey, which assessed the impact of the COVID-19 pandemic on access to health care for people with disability, 56% (n=1375) of respondents reported that regular treatment (eg, physical therapy, dialysis, blood tests) was discontinued owing to the COVID-19 pandemic [6]. Since the outbreak of COVID-19, the rate of emerging health problems and deteriorating health conditions has increased among people with disability (14.7%) to a greater extent than among those without disability (9.9%) [7]. However, the percentage of people with disability (36.8%) receiving medical treatment because of health problems was lower than those without disability (52.5%), and it was found that people with disability had difficulty accessing medical care and using medical services [7]. Especially in the context of the COVID-19 pandemic, individuals with disabilities faced exacerbation of challenges that already existed, encountering barriers such as access to information, communication difficulties, and educational hurdles; however, through the advent of new innovations and strengthened social and familial support, positive transformations are achievable amid the pandemic [8,9]. Therefore, it is essential to provide the latest and easy-to-obtain information on COVID-19 and public health, which is changing rapidly, in a way that suits users [10] and to provide self-management tools using digital health care services.

Mobile apps have emerged as a prevalent modality in personal health care management. As evidenced by 2022 data, approximately 67.1% of the global population engages with mobile phone technology [11]. In the specific context of individuals with disability, a significant surge in smartphone use has been reported, increasing from 54% in the 2012 to 2013 period to a substantial 88% between 2017 and 2018 [12]. A Pew Research Center study revealed that 72% of the population with disability in the United States used smartphones in 2021 [13]. A survey by the National Information Society Agency indicated a slightly lower smartphone use rate of 83.6% in the population with disability in Korea, which is somewhat less than the general population’s 93.5%. However, this still suggests that a significant majority (8 of 10) of individuals with disability use smartphones [14]. At the same time, the number of health management apps that overtake wellness-focused apps has been increasing [15].

Patient-generated health data (PGHD), predominantly sourced from patients via smartphone apps and Internet of Things (IoT) devices, represent an accessible and expansive data collection modality. These apps harness PGHD, enhance health literacy, and position patients as central participants in their health management, thereby catalyzing improved health outcomes [16,17]. From a clinical perspective, PGHD provide unique insights into patients’ day-to-day activities, information typically beyond the purview of standard hospital data acquisition. This additional layer of data offers health care professionals the potential to bolster patient-centric health care strategies and facilitates informed clinical decision-making processes [18]. The growing recognition of PGHD’s importance within health care necessitates expanding digital health services using PGHD. This expansion is crucial for enhancing health equity and ensuring consistent health care access for individuals with disabilities. This study seeks to bolster health care engagement for users with disability by using IoT devices capable of integrating with mobile apps and gathering PGHD, thereby further enhancing health care outcomes.

Cooperation between caregivers and health care professionals is essential for the health care of people with disability. Primary caregiving, often undertaken by families or trusted friends, includes managing daily activities and complex health tasks [19]. Given their roles, caregivers significantly influence the health status of those with disability but also endure physical and mental burdens [20]. The other essential stakeholders are health care professionals. It has been verified that if a patient’s health information is shared through the system, it increases the patient’s understanding and participation, and efficient management is possible [21]. Consequently, this study developed an app- and web-based digital health care service platform that connects with health care professionals not only for people with disability but also for caregivers, promoting mutual health awareness and customized self-care.

Diversity in health apps helps increase access to health care. However, their designs often fail to consider individuals with disability [22]. It is necessary to design and verify a corresponding health care app after identifying the priority needs of people with disability for app development [23]. In the past, the National Rehabilitation Center developed a wellness care mobile app called Daily Healthcare 1.0 for the health care of people with disability [24]. In previous usability evaluations of Daily Healthcare 1.0, there was a high level of interest and curiosity toward the app; however, the overall satisfaction and actual use rates were low. Suggestions such as user-friendly improvement and content supplementation were raised as limitations and complementary points of Daily Healthcare 1.0. This study attempted to design a service based on the user’s needs. In addition, Daily Healthcare 2.0 developed into a platform where people with disability, caregivers, and health care professionals can communicate. Caregivers act as companions for users with disabilities, jointly using the same mobile app integrated with IoT devices for health management. Health care professionals monitor the health outcomes of both individuals with disability and their caregivers via a web system. If necessary, providers can transmit relevant information through the app.

### Aim of This Study

This study ultimately aims to increase health care accessibility and digital health equity for people with disability and their caregivers and to connect with health care professionals for personalized self-care. For this, we intend to reflect the users’ needs from the design and develop a digital health care service platform based on PGHD collected through an app and wearable devices to verify its effectiveness.

## Methods

### Overview

**Figure 1:**
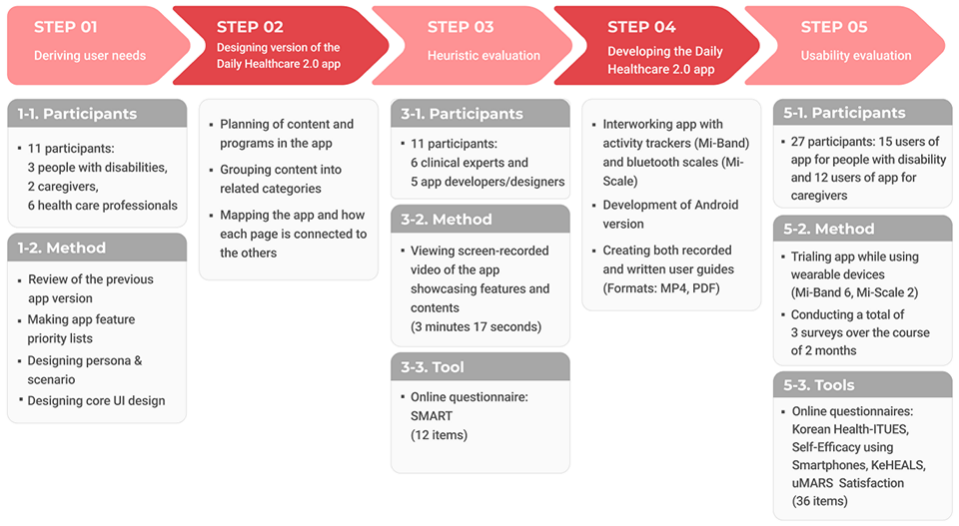
The process of this study. Health-ITUES: Health Information Technology Usability Evaluation Scale; KeHEALS: Korean eHealth Literacy Scale; SMART: smartphone heuristics; uMARS: user version of the Mobile Application Rating Scale.

The progress of this study is illustrated in Figure 1. The Digital Health Equity Framework and Health Equity Impact Assessment emphasize the importance of including vulnerable populations in co-design and evaluation to address their needs in service design for improved digital health equity [25,26]. Therefore, this study began by deriving the needs of people with disability and their caregivers in step 1.

### Step 1: Deriving User Needs

The participants in this study are people with mild physical disability or cerebral lesions such as orthopedic impairment or traumatic brain injury. This was chosen because, as of 2022, out of the 2.65 million people with disability in the country, 53.6% are represented by this group. In this step, people with disability, their caregivers, and health care professionals were recruited from 2 central health and medical centers for people with disability. The recruitment process for research participants was initiated by sending formal documents outlining the study’s objectives to relevant departments across 2 centers within the National Rehabilitation Institute. In response, health care professionals who expressed interest in participating aided in recruiting people with disability and their caregivers.

The importance (1-4 points), validity (1-4 points), and preference (preference: 1; nonpreference: 0) of each app feature in Daily Healthcare 1.0 were evaluated. The highest-scoring features were derived by adding the evaluation scores for each need.

User needs were derived using the following stages: brainstorming and mind mapping, persona and scenario, and developing a user needs list [27,28]. Persona and scenario techniques are the methodologies used to create virtual users of apps developed in this study. These techniques help identify the needs of service users and predict their behavior when implementing the service [29]. First, 2 people were formed into a team for each group (people with disability, caregivers, and health care professionals). Brainstorming and mind maps were conducted on the theme “IoT-connected health care app for those with disability.” Subsequently, through discussion, 1 persona was set up for each team, and a scenario in which the persona uses the health care app was written. The participants then listed the persona’s needs and added them to the checklist. Up to 3 needs were identified through sharing and discussion. When drawing the user interface (UI), the 3 selected user’s needs were included and presented on the UI screen.

### Step 2: Designing Daily Healthcare 2.0

After reviewing the app feature evaluation scores and UI drawn based on the user needs of Daily Healthcare 1.0 in step 1, the priority of app features to be included in Daily Healthcare 2.0 was set. Reflecting on this priority, the content to be included in the Daily Healthcare 2.0 app was planned. Subsequently, the mobile app UI designer composed the menu within the app and designed the UI for the Daily Healthcare 2.0 app.

### Step 3: Heuristic Evaluation for Experts

To identify the expected problems in advance, 11 experts (5 app experts and 6 clinical experts) were recruited. Clinical experts include physicians, nurses, and physical therapists with experience in research or treatment related to people with disability. App experts are app designers or developers with experience in designing or developing health-related apps. Convenience and snowball sampling were used as the recruitment methods [30].

The heuristic evaluation was conducted, assuming that Daily Healthcare 2.0 was implemented. Focusing on the app’s core functions, a link to a 3-minute and 17-second video recording app screen was distributed, and the experts evaluated the app after watching the video guide. Experts who had already been recruited and voluntarily expressed their willingness to participate were sent an email containing links to a video guide and web-based survey. The open web-based survey was created using Survey Monkey (Momentive Global Inc), a specialized web service for web-based questionnaires. Experts who participated in the evaluation were remunerated with an honorarium. Moreover, consultation with an assistive technology expert was conducted to make the app easy to use for persons with disability, along with heuristic evaluations. On the basis of the consultation, the Daily Healthcare 2.0 designer and developer discussed selected factors that should be considered in the development of Daily Healthcare 2.0 for a people with disability–friendly platform.

This study used a short heuristic evaluation tool with 12 questions by Joyce et al [31]. This is the latest heuristic tool, published in 2016, although previous health care app research has used heuristic evaluation tools by Nielsen and Molich [32] or Bertini et al [33], and these traditional tools could be helpful for comparison. In the Joyce et al [31] tool, the top of each item was marked with the word SMART (smartphone heuristics) to differentiate it from other heuristic evaluation tools. The severity rating, which evaluates the severity of the problem for each item, was a 3-point scale (1-3 points) that used the evaluation rating developed by Sauro [34]. It consisted of a 3-point scale to reduce confusion over “0 points” and to collect positive feedback through the “other” item (1: minor; 2: moderate; 3: critical; other: suggestion). The higher the score, the more functional the problem and the more the problem interfered with app use.

### Step 4: Developing Daily Healthcare 2.0

**Figure 2:**
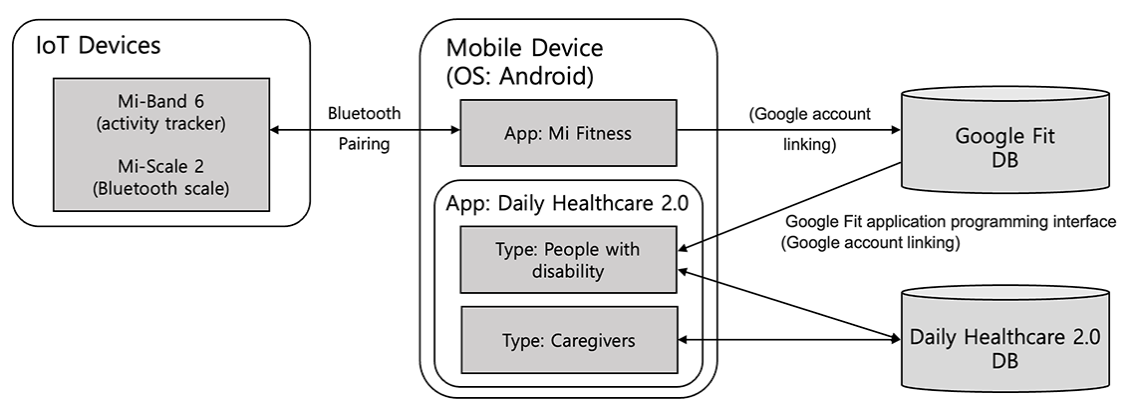
Development of Daily Healthcare 2.0. DB: database; IoT: Internet of Things.

The structure of the Daily Healthcare 2.0 service system is shown in Figure 2. The system was developed into a platform where people with disability, caregivers, and health care professionals can communicate. Caregivers act as companions to users with disabilities, jointly using the Daily Healthcare 2.0 app integrated with IoT devices for their health management. Health care professionals could monitor the health outcomes of both individuals with disabilities and their caregivers via a web system. If necessary, providers can transmit relevant information through the app.

The Daily Healthcare 2.0 app used the Android 12 software development kit and Google Fit application programming interface in the Java environment to collect the PGHD generated during the daily lives of people with disability and their caregivers. An activity tracker (Xiaomi Mi Band 6) and Bluetooth weight scale (Xiaomi Mi-Scale 2) were selected as IoT devices and developed to be connected with the app. Mi Band 6 was used to collect data such as daily walking rate, daily sleep time, and heart rate, and Mi-Scale 2 was used to collect body weight data. PGHD collected by IoT devices were transmitted to Android mobile devices with the Mi Fitness app installed through Bluetooth pairing using the Mi Fitness app. Mi Fitness and Google Fit were linked to the user’s Google account, and finally, PGHD generated by the IoT device were sent to the Google Fit database connected to the user’s Google account. The implemented Daily Healthcare 2.0 app receives PGHD from the Google Fit database to which the user’s Google account is connected through the Google Fit application programming interface, which could be shared and verified by people with disability and their caregivers.

The UI design of the app was modified according to the evaluation results of step 3. In addition, an instructional manual created in PDF and MP4 format was meticulously engineered to augment proficiency in app operation and IoT device integration among people with disability and their caregivers.

### Step 5: Usability Evaluation for App Users

#### Overview

UI or user experience guidelines on mobile app or web accessibility for individuals with disabilities and considerations for development [35,36] emphasize that the UI or user experience design should not necessarily be exclusive to individuals with disabilities. Instead, the design should be universally accessible and usable, including for older people, irrespective of the degree of disability. Hence, this study conducted usability evaluations on the Daily Healthcare 2.0 app not strictly with individuals with disabilities and their caregivers but with a broader demographic to gather meaningful initial feedback.

Daily Healthcare 2.0, strategically designed to enable health information sharing between users with disabilities and caregivers, has attracted user pairs with similar relational dynamics and health management needs. These pairs, including older adult parents with an adult daughter or son, couples, relatives, and friends, exhibited varying degrees of familiarity with the app and IoT device use. For example, parents aged 50 to 70 years requiring technical assistance evaluated the app for people with disability, whereas their tech-savvy offspring (aged 30-40 years) appraised the app for caregivers.

The participants engaged with the IoT devices integrated with the app for 2 months. Usability evaluation was performed 3 times: before, during, and after the intervention. Participants who had already been recruited and expressed their willingness to participate in the study underwent 3 usability evaluations administered through web-based surveys. The open web-based surveys were created using Survey Monkey, a specialized web service for web-based questionnaires. Each time a usability evaluation was conducted, a link to the web-based survey was sent to the participants’ email addresses, encouraging voluntary participation. Upon completing the survey, participants were compensated with a nominal participation fee. All questionnaires were consistent in their composition. After the questionnaire, the participants were checked for their consent to the in-depth interview. The interview was conducted as a 1:1 semistructured interview over the phone by organizing questions based on the questionnaire used in the usability evaluation and about their experience (Multimedia Appendix 1). No usability evaluation has been conducted on the web interface, designated “Wellnesscare,” developed for health care professionals. This interface merely represents an upgrade intended for interoperability with the previously implemented disability management system [37] and Daily Healthcare 2.0 app. Wellnesscare allows health care professionals to register the individuals with disability they supervise, allowing them to monitor their activity records effectively.

To enhance digital health equity, improvements in technology and internet access, as well as literacy related to the technology, are required [2]. This study measured app improvements using the Korean Health Information Technology Usability Evaluation Scale (Health-ITUES), and literacy was assessed using the Korean eHealth Literacy Scale (KeHEALS). Smartphone self-efficacy and satisfaction with app use were also measured. The questionnaire contained 36 items. There were 20 items on health IT usability, 4 on smartphone self-efficacy, 8 on eHealth literacy, and 4 on satisfaction. Each item was evaluated using a Likert scale with responses ranging from “strongly agree” (5 points) to “strongly disagree” (1 point).

#### Health IT Usability

The Health-ITUES, previously deployed among adult users and medical professionals [38], was used in its Korean adaptation, the Korean Health-ITUES, as translated by Lee and Schnall [39]. The Health-ITUES is structured around four subdomains: (1) impact, (2) perceived usefulness, (3) perceived ease of use, and (4) user control. In the research conducted by Lee and Schnall [39], Cronbach α coefficient was determined to be .951, whereas in this study, it was found to be .967.

#### Smartphone Self-Efficacy

In this study, the measure of smartphone self-efficacy was adapted from the digital media literacy assessment instrument developed by Kim [40] and modified to fulfill our research objectives. In the investigation by Kim [40], the reported Cronbach α value was .901, whereas in this study, it was .906.

#### KeHEALS Evaluation

Digital literacy was measured in this study as a key factor in reducing the digital divide and improving digital health equity. KeHEALS was used to measure the participants’ ability to search for, comprehend, and evaluate desired health information from the internet, enabling them to address health issues. KeHEALS leverages a culturally adapted Korean version of the eHealth Literacy Scale [41], which was initially devised by Norman and Skinner [42]. In the research by Chang et al [41], the reported Cronbach αvalue was .89, whereas in this study, it was .958.

#### Satisfaction

In this study, satisfaction was assessed using subjective evaluation items from the user version of the Mobile Application Rating Scale [43], a tool developed to evaluate the quality of apps [44].

This tool includes questions pertaining to anticipated future use frequency, willingness to recommend, and willingness to pay alongside satisfaction measurements. The inquiries for this assessment were used in their Korean-translated version [45] to ensure relevance and comprehension in the target demographic. In the research by Stoyanov et al [43], the reported Cronbach α value was .78; in this study, it was .743.

### Ethical Considerations

The study design was approved by the institutional review board of Seoul National University Hospital (C-2109-136-1257). Before conducting the study, all participants were provided with an explanatory document, and after obtaining their informed consent, needs were derived and evaluations were performed.

All research-related data were collected solely for this study. All information gathered from the participants was securely stored in encrypted files, accessible only to the principal investigator and authorized research team members. The converted research data files are stored in a cloud database held by the principal investigator, and access is restricted to approved research team members.

### Data Analysis

Health IT usability, smartphone self-efficacy, KeHEALS, and satisfaction were evaluated using a Likert scale ranging from 1 to 5. This study used descriptive statistics to assess the significance of variations in the measured values before, during, and after the usability evaluation, using mean and SD as the metrics of interest. During the focus group interviews, significant points were noted down by the research team. Themes were used to categorize the collected interview data through discussions among the researchers.

## Results

Funding was provided for research for 8 months in 2021, and 49 individuals with disabilities, caregivers, health care professionals, and informatics experts participated until November 2021. In addition, data analysis was conducted until May 2022.

### Step 1: Deriving User Needs

A total of 11 participants (3 individuals with disability, 2 caregivers, and 6 health care professionals) were evaluated on 19 app features in Daily Healthcare 1.0 according to their needs. As a result, “guide to welfare benefits for the disabled” received the highest score with 76 points, followed by “provide information on health behavior guidelines for the disabled in national disaster situation (COVID-19)” and “information on health examination methods and institutions for the disabled” with 72 points, and “provide educational contents” with 71 points. These 4 needs items were “favored” by 10 of 11 participants.

Two teams comprised individuals with disability (among the 3 people with disability, 1 individual had difficulty with the speed of conversation and using their hands, so with the assistance of a researcher, needs were derived for that individual alone), 1 team of caregivers, and 3 teams of health care professionals engaged in brainstorming and mind mapping regarding the Disability IoT App. Following the sessions, each team created one persona and a corresponding use scenario for the app.

Four common needs were identified from the persona’s scenarios of the people with disability team: “guide to welfare benefits for the people with disability,” “provide educational content,” “provide information on health behavior guidelines for the people with disability in national disaster situations (COVID-19),” and “telemedicine.” Furthermore, additional needs were identified, including “link to first aid services in case of an emergency,” “creating a community,” “guide to health examination institutions for the people with disability,” and “booking means of transportation.” In the caregiver team’s persona scenario, a need for “training content related to exercise or nutrition” was identified. For the health care professional team, needs such as “institution/service linkage and monitoring,” “guide to welfare benefits for the disabled,” “provide information on health behavior guidelines for the people with disability in national disaster situations (COVID-19),” “provide health monitoring and feedback,” and “provide a PGHD sharing platform for the people with disability and caregivers” were derived. Examples of the identified personas and scenarios are presented in Textbox 1.

**Examples of the identified personas and scenarios.**

**Persona: people with disability team A**

• Occupation: employee at a disability center

• Education level: graduate of a vocational college

• Annual salary: 30 million KRW (US $22,188)

• Persona quote: “Let’s live without being subservient. Let’s not give up!”

• Web-based activity: actively engaged

• Offline activity: frequently meets friends who are colleagues at work, gradually meets fewer past friends due to changes in lifestyle, participates in disability-related rallies

• Entry point: easily obtains health management app information at the disability center

• Social media proficiency: uses social media 1 to 2 times a day, frequently uses Facebook, utilizes YouTube for video consumption

• Smartphone proficiency: high

• Technological proficiency: high, adept at using a laptop

• Customer goals: acquiring information, sharing with others, utilizing disability-related information for work purposes at the disability center

• Desired information or features: welfare benefits, health check-ups, medication-related information, etc.

**Scenario: health care professionals team A**

• Sumi is responsible for the health management of an intellectually disabled individual with diabetes who is caring for a newborn. She arrived at the office of Health Center in the morning and powered on her computer. An alert notified her that the individual did not attend their outpatient visit for diabetes management yesterday. Sumi contacted the individual to inquire about the reason but realized they lacked a clear understanding of the importance of diabetes management. Through the Wellnesscare system, Sumi sent a link to the individual’s disability-specific app, containing a video titled “Understanding Diabetes and Health Management,” to enhance their understanding of diabetes. Additionally, she adjusted the upcoming outpatient schedule and informed the individual about the revised schedule. Sumi also uploaded the outpatient schedule information to an information-sharing application to enable mutual information exchange.

**Figure 3:**
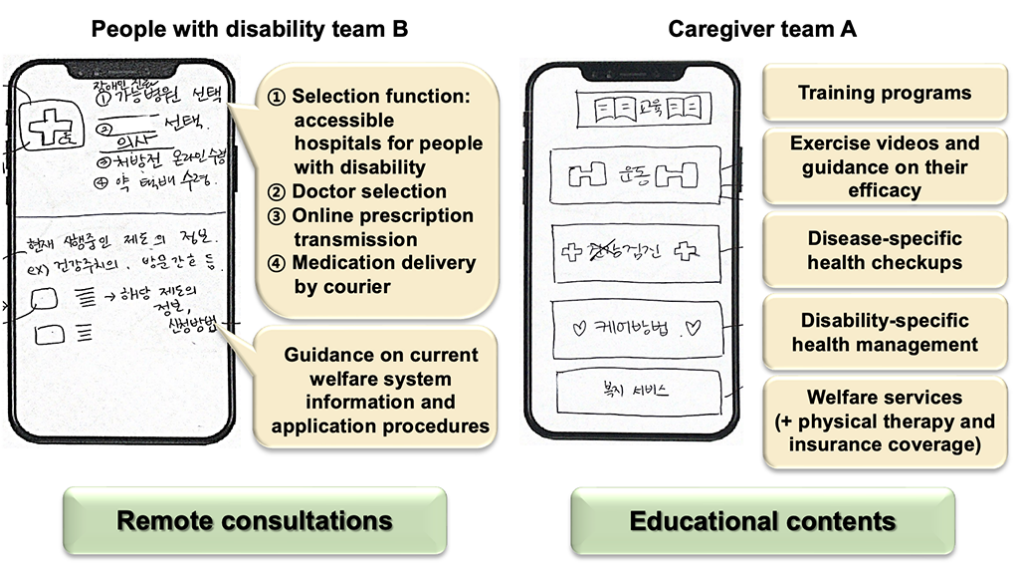
App user interface created by the participants.

Participants created app UIs implementing high-priority needs identified from the personas’ scenarios. Six UIs were developed in the disabled team, 6 UIs were developed, including 2 for “welfare benefits information,” 2 for “remote consultations,” 1 for “educational content,” and 1 for “examination methods and facility guidance.” The caregiver team designed 3 UIs associated with “educational content,” “accessing nutrition/diet information,” and “health monitoring and feedback.” The health care professional teams produced 5 UIs related to “welfare benefits information,” “educational content,” “health information sharing,” “remote services,” and “disability health behavior guidelines during disasters.” Among the total of 14 UIs, the most frequently depicted needs were “disability welfare benefit information” (3 UIs) and “provision of educational content” (3 UIs). Examples of the UIs created by the participants are illustrated in Figure 3.

### Step 2: Designing Daily Healthcare 2.0

In step 1, the feasibility of developing the UI and high-priority needs derived from the personas were assessed, and alternative methods of providing information were considered if development was not feasible. Consequently, the Daily Healthcare 2.0 app features were organized as follows: home, profile, health records, health record review, wellness assistant (educational content and information provision), health together (information sharing with caregivers), and community. In addition, the app incorporated the visualization of PGHD collected from IoT devices in graphs and included a feature for users to self-record meals and medication information separately. Furthermore, the app allowed users to set goals for healthy activities such as alcohol consumption, smoking, and exercise, and track the progress toward achieving those goals. The design process prioritized the app’s development for users with disabilities, which served as the foundation for designing the app for caregivers.

### Step 3: Heuristic Evaluation for Experts

The heuristic evaluation, conducted with 11 experts (6 clinical experts and 5 app experts), revealed that usability issues within the app were assessed to be minor, closely approaching a score of 1 on a severity scale (12 items: mean 1.37, SD 0.31). Among these items, the “SMART 3: prevent problems and resolve problems immediately” item received the highest average score of 1.91 (SD 0.79), whereas the “SMART 5: focus on one task” category received the lowest average score of 1.09 (SD 0.29).

The opinions of 11 expert evaluators are presented in Table 1. The following features have been added and improved for users with disabilities or older people: high-contrast color design, text size enlargement function, automatic log-in features, detailed color specifications for each function, and insertion of the main menu at the top of the menu tree. In addition, the health records and health record verification menus were integrated to streamline the main menu.

**Table 1:** Opinions gathered through expert-targeted heuristic evaluation (n=11).

SMART^a^ items	Opinions	Whether to apply
**SMART 1: Provides immediate notification of status**	App push notifications limited to a maximum of 2 times Send a reminder immediately that must be checked Extended duration of error message pop-ups considering users with disability or older adults Message alert for log-in failures	Apply
**SMART 2: Use consistent terms with familiar rules and standards**	Auto-refresh feature needed when scrolling up or down for convenience Adjust spacing between main buttons at the bottom Need a concise layout for the main screen Simplify menu names	Apply
**SMART 3: Prevent problems and resolve problems immediately**	Automatic detection and connection of IoT^b^ devices via Bluetooth In-app inquiry feature or connection to available inquiry channels	Apply
**SMART 4: Show overlay for key features**	Need for implementation of coaching marks considering disabilities and older adults Addition of the mentioned feature to the basic guide or tutorial section	Partially apply (tutorial enhancement needed)
**SMART 5: Focus on 1 task**	Suggested application of visual feedback for task performance in apps for individuals with disability	Apply
**SMART 6: Visual interface design**	Notification of inconvenience when optimization for various resolutions is not applicable Maintaining consistency through uniformity of button and top bar colors Design modifications for individual items to prevent confusion	Apply
**SMART 7: Intuitive interface**	Displaying pictorial icons to enhance UI^c^ accessibility	Apply
**SMART 8: Clear path to search**	Clear guidance on the process of integrating with IoT devices is necessary	Apply
**SMART 9: Allow configuration options and shortcuts**	Addition of a feature to allow users to customize the placement of their most relevant feature at the top of the home screen Enhanced visibility and emphasis on the crucial Add Disabled Individual/Caregiver Account button across various pages	Partially apply (home screen enhancement needed)
**SMART 10: Support for a wide range of mobile environments**	Implementation outside of Android	Do not apply (planned)
**SMART 11: Ease of input**	Clear separation of dropdown and text areas Increase ease with text prediction Enhanced usability through features such as text prediction Necessity for the consideration of adaptive access for facilitating app accessibility for users with disabilities Clear distinction between dropdown and text fields Need to think about alternative input (adaptive access) for individuals with disability to access apps	Partially apply (discussion on alternative access)
**SMART 12: Use of cameras, microphones, and sensors**	Currently, the app does not implement the features	Do not apply (discussion on the app)

^a^SMART: smartphone heuristics.

^b^IoT: Internet of Things.

^c^UI: user interface.

### Step 4: Developing Daily Healthcare 2.0

**Figure 4:**
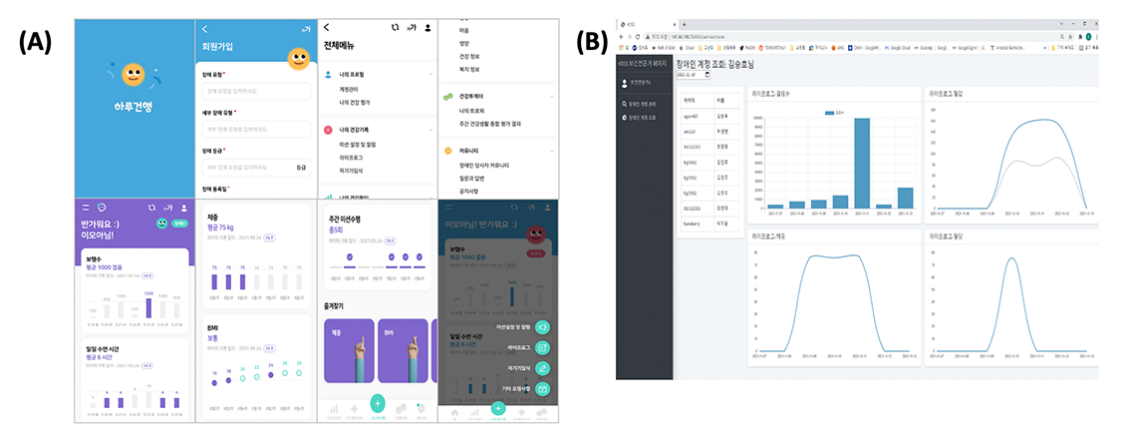
Screens of (A) Daily Healthcare 2.0 app and (B) Wellnesscare web.

The Daily Healthcare 2.0 app was developed by integrating not only the results of expert-oriented heuristic evaluations but also perspectives from various fields, including guidelines for app development targeting individuals with disabilities, as well as app developers and UI designers. The Wellnesscare website for healthcare professionals has been updated to recommend personalized health management content based on the user’s health data. On the website, they can access health records and educational content, including the PGHD of users with disability and their caregivers, to provide comprehensive evaluations of weekly health behaviors. The screens of the Daily Healthcare 2.0 app and Wellnesscare website are illustrated in Figure 4.

**Figure 5:**
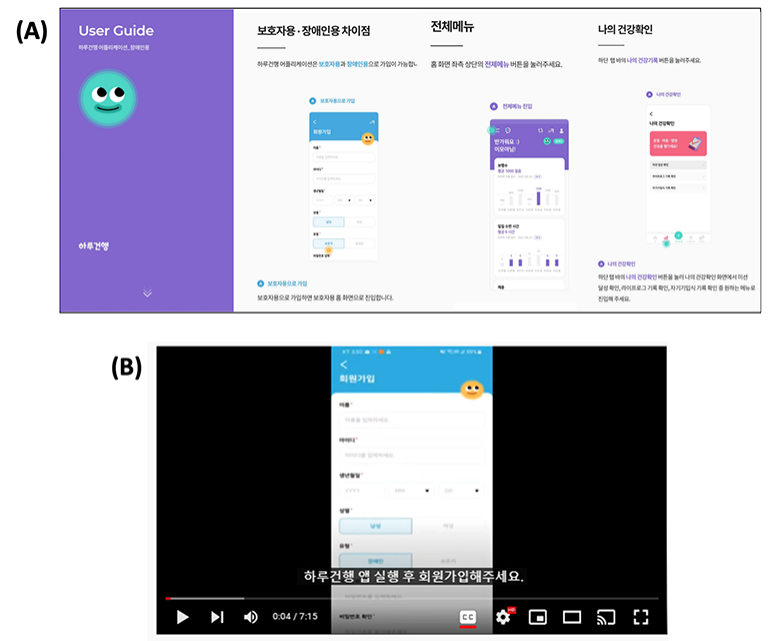
Screens of Daily Healthcare 2.0 user manual: (A) PDF version and (B) MP4 version.

To enhance app usability and user compliance, user manuals for both the app designed for individuals with disability and the app designed for caregivers were developed. The manuals were created in 2 formats: PDF and MP4. They provide step-by-step instructions, accompanied by illustrations and written guidance for all app functionalities, including app installation, IoT device integration, self-recording and verification of PGHD, and access to health information for individuals with disabilities and caregivers. Figure 5 shows a section of the manual.

### Step 5: Usability Evaluation for App Users

#### Overview

A total of 27 participants (15 app users of people with disability and 12 caregiver app users) completed the usability evaluation. Although we initially recruited 15 participants for the app for people with disability and 15 participants for the caregiver app, 3 caregiver app participants who did not actively engage in the program for more than 5 days were excluded from the analysis of the results (Tables 2 and 3).

**Table 2:** General characteristics of usability evaluation (n=27).

Characteristics		App for people with disability	App for caregivers
Age (years), mean (SD)		50.7 (14.0)	41.3 (12.4)
Age group (years), n (%)			
	20-39	6 (40)	8 (67)
	40-59	1 (7)	2 (17)
	≥60	8 (53)	2 (17)
**Sex, n (%)**			
	Male	8 (53)	5 (42)
	Female	7 (47)	7 (58)

**Table 3:** Scores for usability evaluation (n=27).

Usability evaluation	Scores, mean (SD)					
	Prequestionnaire	Midquestionnaire	Postquestionnaire	Prequestionnaire	Midquestionnaire	Postquestionnaire
Health IT usability	3.7 (0.7)	3.5 (0.6)	3.7 (0.7)	2.2 (1.1)	3.5 (0.7)	3.8 (0.7)
Smartphone self-efficacy	3.7 (0.8)	3.6 (0.8)	3.8 (0.5)	2.8 (1.7)	3.6 (0.8)	4.4 (0.6)
eHealth literacy	3.5 (0.6)	3.6 (0.7)	3.7 (0.7)	2.9 (1.7)	3.7 (1.1)	3.9 (1.1)
Satisfaction	2.5 (0.8)	2.9 (0.8)	3.9 (1.2)	3.4 (0.8)	3.1 (1.1)	4.0 (0.9)
Willingness to recommend	3.2 (1.1)	3.4 (1.2)	3.7 (0.8)	3.1 (0.9)	3.5 (0.9)	3.7 (1.2)
Willingness to pay	3.9 (1.4)	3.8 (1.3)	2.9 (1.2)	2.0 (1.0)	4.0 (0.9)	2.2 (1.3)

**Figure 6:**
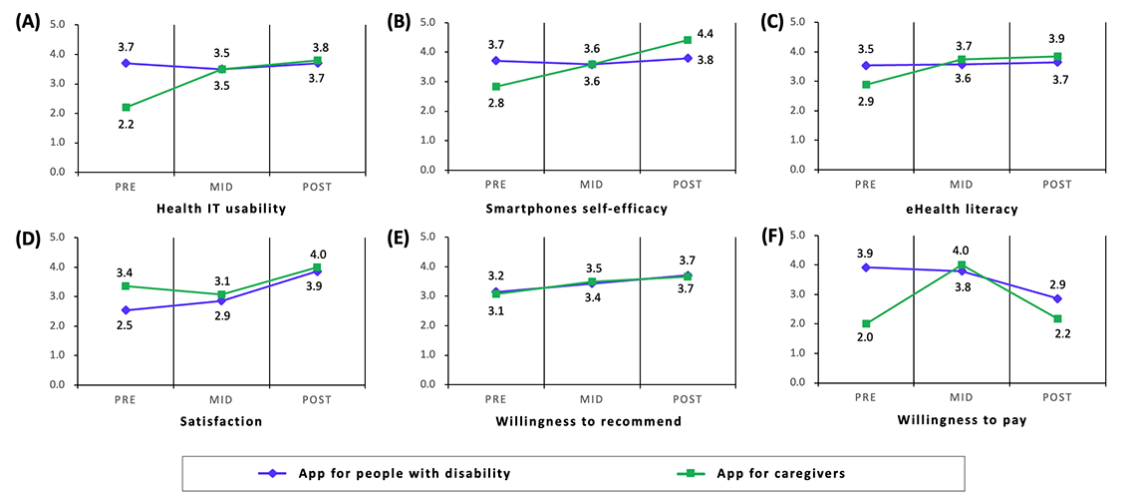
Results of usability evaluation: (A) health IT usability, (B) smartphone self-efficacy, (C) eHealth literacy, (D) satisfaction, (E) willingness to recommend, and (F) willingness to pay.

After using the Daily Healthcare 2.0 app, both users of the app for people with disability and users of the app for caregivers experienced an increase in health IT usability, smartphone self-efficacy, eHealth literacy, and satisfaction. The willingness to recommend also increased. However, the willingness to pay decreased (Table 2). Furthermore, an additional analysis was conducted to examine the impact of age on app use by dividing the participants into groups based on age, with 60 years as the cutoff point (Figure 6).

The results of in-depth interviews conducted with 13 participants who agreed to participate (6 users of app for people with disability and 7 caregiver app users) and representative quotes are shown below.

#### UI and Health Graphs

The participants said the health graphs were very helpful.

[With the app], my health data is visually displayed in graphs, showing the trends over time. Using the app made me more conscious about my health. Seeing these health issues visually on the app made me realize that I need to be cautious.

User A of app for people with disability

To be honest, I tend to forget records as time passes. However, the app accumulates all the records and presents them in graphs, which I find beneficial in showing the trends.

User D of app for people with disability

The app’s intuitive and streamlined design allowed for easy familiarization with its features, even for first-time users.

User C of caregiver app

#### Providing Health Information and Exercise Video Content

Three users mentioned that health information and exercise video content was useful.

Information related to diabetes or blood pressure-related conditions was found to be most helpful. Specifically, information on how to manage meals was highly beneficial.

User B of app for people with disability

It is difficult for me to distinguish which exercise videos, like those on YouTube, are helpful. However, in this app, experts recommend them, which increases my trust in their reliability.

User A of caregiver app

Since experts recommend the exercise videos, I think they will be particularly helpful for people like me who are new to exercising.

User C of app for people with disability

#### Caregiver Integration Function

One user said the caregiver integration function was beneficial.

Caregiver app user A: “Since there is no guarantee that the caregiver and the disabled individual living in the same household, the data integration feature is beneficial. It would also be good to know the health status of the disabled individual. Having the alarm function to the caregiver would be even better.”

#### User Manual

Having two types of manuals was useful.

The availability of a manual proved beneficial in acquiring proficiency with the functions. Particularly for elderly users, who may find reading text challenging, providing accompanying videos of appropriate duration (approximately 7 minutes) was helpful.

User F of caregiver app

#### Willingness to Pay (Additional Question)

In assessing the willingness to pay for the app’s services, several users shared their perspectives on the value proposition and potential improvements.

Most users’ concept of value does not seem to include information. However, I would be willing to pay a fee if the service had national-level and highly trustworthy expert management. However, since this app is currently in its early stages, I am unwilling to pay for it.

User A of app for people with disability

While it is great that it is an app from the National Rehabilitation Center for the health of the citizens, I believe there should be experts who review my data and provide feedback to justify the cost.

User A of caregiver app

Currently, I perceive this app as managing daily life rather than addressing health discomfort, so I am unwilling to pay. However, I would be willing to pay for it if the app had the effect (such as digital therapeutics), such as medication or clinical treatment.

User F of caregiver app

## Discussion

### Principal Findings

In this study, we developed a digital health care service platform that connects individuals with disability, caregivers, and health care professionals to enhance digital health equity. To accomplish our objectives, we developed the Daily Healthcare 2.0 app, tailored for individuals with disability and their caregivers. This app was designed based on user needs and facilitated the sharing of PGHD via both the app and IoT devices. Concurrently, the Wellnesscare web platform allows health care professionals to monitor user data. Following a heuristic evaluation and subsequent usability evaluation, our integrated health care service platform, encompassing both Daily Healthcare 2.0 and Wellnesscare, demonstrated enhancements in users’ eHealth literacy, smartphone self-efficacy, satisfaction, and propensity to recommend. However, there was a noted decline in their willingness to pay.

### Comparison With Prior Work

The findings of this study align with those of previous research that highlights the correlation between enhanced digital literacy and the ability of patients and caregivers to perform role-specific functions in a digital environment, exchange health information with health care professionals through web-based platforms, and actively engage in the co-design and delivery of health care services [46]. Furthermore, digital literacy signifies the capacity to provide health promotion services within a digitally inclusive environment that ensures equitable and nondiscriminatory health care access based on patient needs [47]. This means that users with increased literacy scores will contribute to digital health equity and facilitate the transition toward a human-centered approach in delivering health services. Given that a national rehabilitation center led this platform research, it is crucial to continue efforts to enhance the capabilities of individuals with disability in exploring digital health care environments and to foster collaborative cocreation to promote the shared values of health and digital health equity [48].

Active user involvement in the design phase positively affects system success and user satisfaction [49]. Moreover, it also contributes to enhancing users’ eHealth literacy [50]. In this study, incorporating diverse user needs from the design phase was identified as one of the strategies that positively evaluated the platform’s effectiveness. The needs of users with disability, caregivers, and health care professionals prioritized providing welfare benefits information and educational content related to disability. This was likely due to the difficulty in finding information on specific steps, such as selecting suitable benefits and reliable information, even though national disability welfare information websites exist. Health care professionals also reported the lack of appropriate communication channels, excluding face-to-face interactions, to deliver welfare benefits or educational materials to individuals with disabilities and the caregivers they are responsible for. Especially recently, face-to-face communication has become even more challenging owing to the COVID-19 pandemic. As a result, the need for information channels applicable during national emergencies (such as the COVID-19 pandemic) and the provision of health behavior guidelines for individuals with disability have emerged as high priorities. These findings indicate that users desire communication channels to exchange reliable information related to health, going beyond simply recording and monitoring the current health status in digital health care services, reflecting the current social situation (the COVID-19 pandemic). The Daily Healthcare 2.0 and Wellnesscare systems are significant as they connect individuals with disability requiring health care management, caregivers, and health care professionals, providing a convenient platform for suitable and reliable health information. This enables individuals to receive professional assistance through mobile apps, even at home.

The service system developed in this study attempted to establish connections among individuals with disability, caregivers who play a crucial role in their health care management, and health care professionals as active users. Various studies have focused on health care management apps with a clinical focus, such as medication scheduling and skin integrity education programs, aiming to alleviate caregiver burden [51], connect individuals with disability and caregivers [52,53], or develop platforms that connect individuals with disabilities, caregivers, and health care professionals. However, these studies primarily focused on clinical support or system development [54]. In contrast, in our study, all 3 groups were actively involved as users, and the platform was designed to establish a bilateral linkage of PGHD between individuals with disability and caregivers. This approach is highly valuable, as stated by 1 participant, as Daily Healthcare 2.0 can serve as a partner, allowing individuals with disability and caregivers to manage their health together, even if they do not reside together. Furthermore, the Daily Health care 2.0 system has positively improved digital health equity by assisting users with disability and their caregivers in health care management, particularly in nonhospital settings where health care management may be neglected.

Interestingly, although the service platform received mostly positive evaluations, willingness to pay for the service decreased. Additional interviews were conducted to investigate the reasons for this. The results revealed that users cited several reasons for their lack of willingness to pay. They mentioned that the service did not provide personalized feedback, only offered monitoring without tailored recommendations, and lacked credibility because it was in the early development stage. These findings align with previous research indicating that the involvement of health care professionals enhances user health care management effectiveness and that health care app users desire feedback from professionals [55,56]. In addition, the involvement of health care professionals improves user engagement and loyalty to health care apps [49].

### Strengths and Limitations

PGHD from individuals with disability and caregivers were used to motivate and enhance the effectiveness of health care management. PGHD promote self-management outside of health care settings and communication with health care professionals [57]. The need for a platform to share PGHD among users with disabilities and their caregivers was identified through the needs assessment conducted in this study. To facilitate a diverse PGHD collection, we used user-friendly and highly functional wearable devices, such as wristbands and Bluetooth-enabled weighing scales. The PGHD generated from individuals with disabilities and their caregivers through these IoT devices were designed to be integrated with the mobile app and shared not only between users but also with health care professionals who can provide advice on health matters. Therefore, this study serves as a significant validation of the use of PGHD from not only mobile devices but also popular IoT devices in community-based health management of individuals with disability.

Despite the significant findings of this study, there are several limitations. First, this study involved participants without disability in the usability evaluation, although the platform was designed for individuals with disability and their caregivers. To address this limitation and adhere to the Guidelines for Development for Persons with Disabilities, which state that apps should apply to all users, including individuals with disabilities, users with relatively lower mobile familiarity or literacy were assigned as the user group for the disability app. Nonetheless, it is recommended that future studies be conducted with actual individuals with disability and caregivers for further robust evidence. Second, technical improvements are necessary. The Daily Healthcare 2.0 app shares all user-generated PGHD without any screening. However, considering privacy protection and preferences, it is proposed to incorporate a feature that allows users to activate information sharing for each PGHD item selectively. Third, it is essential to note that the Daily Healthcare 2.0 app was developed for the Android platform, and the use of IoT devices was limited. Therefore, these limitations must be overcome to fully use this system in real-world community settings.

### Future Directions

In this study, users expressed high satisfaction with the structure of the platform, particularly the Wellnesscare web features that allow health care professionals to monitor users’ health records. However, users also expressed a desire for more personalized and immediate feedback from experts, beyond just monitoring. Future research should consider incorporating personalized exercise videos or welfare benefits information recommended by algorithms based on expert reviews and users’ PGHD.

## Appendix

Multimedia Appendix 1 Interview guide.

## Abbreviations

Health-ITUES: Health Information Technology Usability Evaluation Scale
IoT: Internet of Things
KeHEALS: Korean eHealth Literacy Scale
PGHD: patient-generated health data
SMART: smartphone heuristics
UI: user interface

## Data Availability

Data from this study are available upon reasonable request from the corresponding author.
