# Supplementary material for: Digital Health Equity and Tailored Health Care Service for People With Disability: User-Centered Design and Usability Study
Source: J Med Internet Res. 2023 Nov 28;25:e50029. doi: 10.2196/50029 (PMC10716768; doi:10.2196/50029)
Supplement: Multimedia Appendix 1 [file jmir_v25i1e50029_app1.docx]

## Appendix 1: Interview Guide

Interview Questions:

1. How frequently did you use the app during the research participation period?

1-1. Overall, did you find the app user-friendly?

1-2. Was it easy to familiarize yourself with the app's features?

1. Did the app assist in your health management? (Improvement in health management or quality of life)

2-1. Which feature was most helpful for health management?

2-2. Did you receive adequate information regarding health management?

2-3. Is there any additional information you would like to see in the app?

1. Was the feature linking the app for individuals with disabilities and caregivers helpful for health management?
2. How satisfied are you with the health management program provided by the app?
3. Were there any inconveniences or areas for improvement while using the app?
